# βTrCP controls the lysosome-mediated degradation of CDK1, whose accumulation correlates with tumor malignancy

**DOI:** 10.18632/oncotarget.2274

**Published:** 2014-07-27

**Authors:** Joaquín Herrero-Ruiz, Mar Mora-Santos, Servando Giráldez, Carmen Sáez, Miguel Á. Japón, Maria Tortolero, Francisco Romero

**Affiliations:** ^1^ Departamento de Microbiología, Facultad de Biología, Universidad de Sevilla, Sevilla, Spain; ^2^ Instituto de Biomedicina de Sevilla (IBIS), Hospital Universitario Virgen del Rocío/CSIC/Universidad de Sevilla and Departamento de Anatomía Patológica, Hospital Universitario Virgen del Rocío, Sevilla, Spain

**Keywords:** apoptosis, autophagy, βTrCP, cancer, CDK1

## Abstract

In mammals, cell cycle progression is controlled by cyclin-dependent kinases, among which CDK1 plays important roles in the regulation of the G2/M transition, G1 progression and G1/S transition. CDK1 is highly regulated by its association to cyclins, phosphorylation and dephosphorylation, changes in subcellular localization, and by direct binding of CDK inhibitor proteins. CDK1 steady-state protein levels are held constant throughout the cell cycle by a coordinated regulation of protein synthesis and degradation. We show that CDK1 is ubiquitinated by the E3 ubiquitin ligase SCF^βTrCP^ and degraded by the lysosome. Furthermore, we found that DNA damage not only triggers the stabilization of inhibitory phosphorylation sites on CDK1 and repression of *CDK1* gene expression, but also regulates βTrCP-induced CDK1 degradation in a cell type-dependent manner. Specifically, treatment with the chemotherapeutic agent doxorubicin in certain cell lines provokes CDK1 degradation and induces apoptosis, whereas in others it inhibits destruction of the protein. These observations raise the possibility that different tumor types, depending on their pathogenic spectrum mutations, may display different sensitivity to βTrCP-induced CDK1 degradation after DNA damage. Finally, we found that CDK1 accumulation in patients’ tumors shows a negative correlation with βTrCP and a positive correlation with the degree of tumor malignancy.

## INTRODUCTION

In mammalian cells, cell cycle progression is regulated by a series of cyclin-dependent kinases (CDKs), among which CDK1 has been primarily implicated in G2/M transition, mainly in association with cyclin B (see [1] for review). However, CDK1 is also able to regulate G1 progress and G1/S transition [2, 3]. Given its essential role in the cell cycle, CDK1 is highly regulated. First, CDK1 requires the association of one cyclin to recognize and phosphorylate its substrates [4]. Second, CDK1 is also regulated by phosphorylation and dephosphorylation and changes in subcellular localization [5, 6]. Phosphorylation at Thr161 increases the complex activity, whereas phosphorylation at Thr14 and Tyr15 leads to inhibition of CDK1. Activating T-loop motif phosphorylation is carried out by CDK-activating kinases, whereas inhibitory kinases WEE1 and MYT1 phosphorylate Thr14 and Tyr15; the CDC25 phosphatase family members counteract this activity [7-9]. Finally, CDK1-cyclin complexes are also governed by direct binding of CDK inhibitor proteins [10]. On the other hand, CDK1 protein levels are held at a constant steady-state level throughout the cell cycle, which is maintained by a coordinated regulation of protein synthesis and degradation [11]. In fact, from G1/S transition to G2/M boundary an active *CDK1* translation occurs accompanied by a concurrent degradation. However, little is known regarding the destruction of CDK1. It has been shown that CDK1 is downregulated under genotoxic stresses, that double-stranded RNA-activated protein kinase (PKR) is involved in the process and that PKR-mediated Tyr4-phosphorylation facilitates CDK1 ubiquitination and proteosomal degradation [12]. Nevertheless, the CDK1-specific E3 ubiquitin ligase remains to be identified. In this study we found that CDK1 is ubiquitinated by SCF^βTrCP^ and degraded by the lysosome. Furthermore, we analyzed the effect of DNA damage on CDK1 stability and on induction of apoptosis. Finally, we found that CDK1 accumulation in patients’ tumors shows a negative correlation with βTrCP levels and a positive correlation with the degree of tumor malignancy.

## RESULTS

### βTrCP binds to CDK1

**Figure 1:**
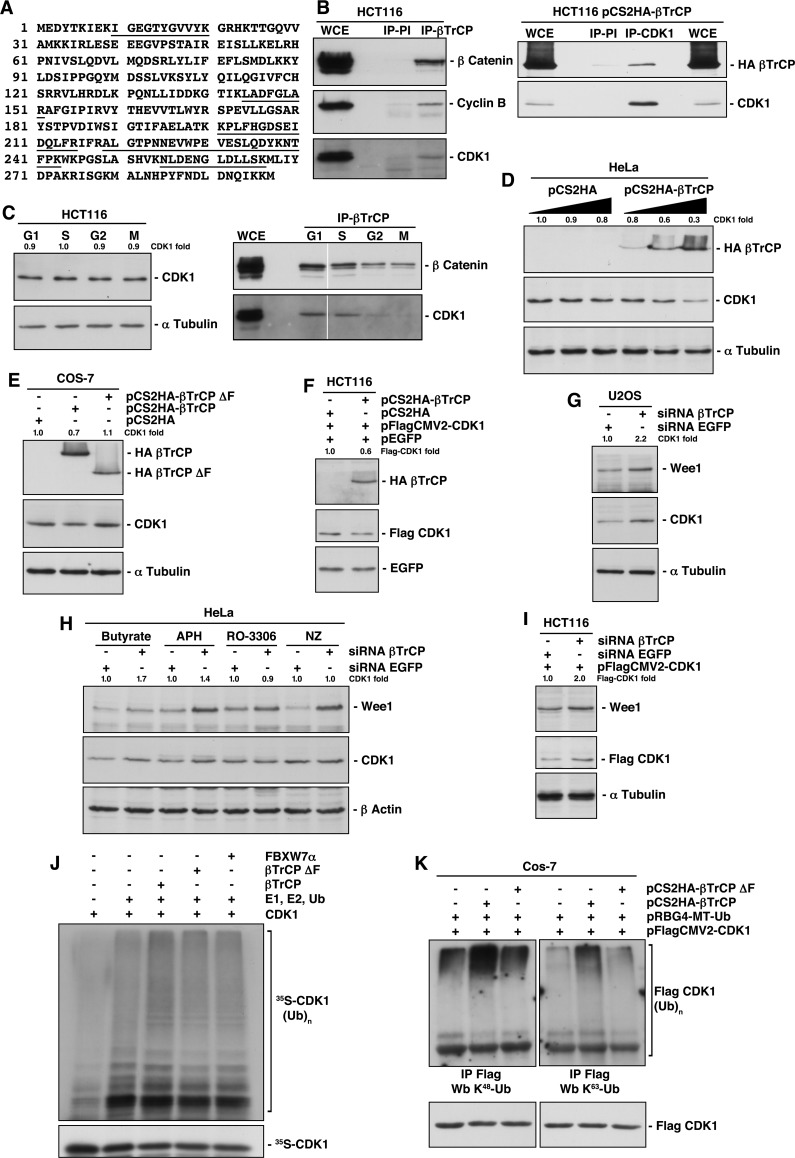
Identification of CDK1 as a new substrate of SCF^βTrCP^ (A) Five peptides of CDK1, sequences underlined, were observed by MS/MS. (B) Whole cell extracts (WCE) from HCT116 or HCT116 transfected cells were used to immunoprecipitate βTrCP (left panel) or CDK1 (right panel), and complexes were analyzed by immunoblotting. IP-PI: immunoprecipitation with normal rabbit (left panel) or mouse (right panel) sera. (C) HCT116 cells were synchronized in the different phases of the cell cycle as described in Methods, and analyzed by Western blot (left panel) or by immunoprecipitation (right panel). (D, E, F) HeLa, Cos-7 or HCT116 cells were transfected with the indicated plasmids and analyzed by immunoblot. (G) U2OS cells were interfered with the indicated siRNAs and analyzed by Western blot. (H) HeLa cells were interfered with the indicated siRNAs, and 24 hours before harvesting were synchronized using different drugs: butyrate (G1 phase), aphidicolin (APH, S phase), RO-3306 (G2 phase) or nocodazole (NZ, M phase). Extracts were blotted with the indicated antibodies. (I) HCT116 cells were transfected with pFlagCMV2-CDK1 and interfered with the indicated siRNAs, and analyzed by Western blot. (J) *In vitro* ubiquitin ligation assay of ^35^S labeled *in vitro*-transcribed/translated CDK1 was conducted in the presence or absence of the following products: cold *in vitro*-transcribed/translated βTrCP, βTrCPΔF or FBXW7α, E1 (His_6_-ubiquitin activating enzyme), E2 (His_6_-UbcH3 and UbcH5a) and Ub (ubiquitin). Samples were incubated at 30°C for 1h. The bracket on the right side marks a ladder of bands corresponding to poly-ubiquitinated CDK1. (K) Cos-7 cells were transfected with the indicated plasmids and poly-ubiquitinated Flag-CDK1 visualized after Western blot of Flag immunoprecipitations. Anti-K^48^-Ub and K^63^-Ub polyclonal antibodies recognize K48- and K63-linkage specific ubiquitination, respectively. The quantitative fold change in CDK1 or Flag-CDK1 was determined relative to the loading control.

To find new SCF^βTrCP^ substrates, we searched for novel βTrCP-interacting proteins. To address this, we performed anti-HA immunoprecipitation experiments of nuclear and cytosolic fractions from HA-βTrCP transfected Cos-7 cells, followed by tandem mass spectrometry (MS/MS). Immunoprecipitation reactions using normal mouse IgG served as negative controls. We detected CDK1 in cytosolic immunoprecipitates with 23.9% sequence coverage. Specifically, five different tryptic peptides (Fig 1A) were observed via MS/MS. To further validate the authenticity of this result, we confirmed the presence of CDK1 within the endogenous βTrCP immunocomplex using Western blotting analysis (Fig 1B). Moreover, we also found cyclin B in the immunoprecipitation, which was also identified in the proteomic analysis with a single peptide. Anti-β-catenin was used as an internal control since commercial anti-βTrCP is not available for Western blot. In addition, we performed reciprocal immunoprecipitations using anti-CDK1 monoclonal antibodies and, as shown in figure 1B, CDK1 was also able to precipitate HA-βTrCP. Furthermore, we studied at which phase the interaction with βTrCP occurs, as CDK1 protein is present in all phases of the cell cycle. Figure 1C shows that βTrCP binds to CDK1 in G1 and S phases of the cell cycle.

### Expression of βTrCP regulates CDK1 stability

Next, we analyzed the effect of βTrCP over- and underexpression on CDK1 levels. We found that increasing amounts of pCS2HA-βTrCP progressively reduced levels of endogenous CDK1 (Fig 1D). Furthermore, a βTrCP mutant lacking the F-box domain (βTrCPΔF) suppressed the CDK1 diminution (Fig 1E), involving full ubiquitin ligase in this scenario. Similar results were obtained when we explored this effect on a constitutively expressed CDK1, avoiding the potential transcriptional consequences of the βTrCP overexpression (Fig 1F). Reciprocal experiments were carried out using knockdown experiments. The efficiency of siRNA-βTrCP was verified interfering pCS2HA-βTrCP transfected HeLa cells by testing HA-βTrCP depletion (supplementary Fig S1A). We then interfered several cell lines and observed an increase of CDK1 upon βTrCP depletion, similar to that achieved for WEE1, a known substrate of SCF^βTrCP^ (Figs 1G-1I), and a longer half-life of the protein (supplementary Fig S1B). This increase was only perceived in G1 and S phases (Fig 1H), precisely where the βTrCP/CDK1 interaction is detected. Likewise, βTrCP depletion also augmented transfected Flag-CDK1 (Fig 1I and supplementary Fig S1C), ratifying the post-translational effect of βTrCP expression on CDK1. Together, these results reveal that βTrCP regulates CDK1 stability.

### SCF^βTrCP^ ubiquitinates CDK1 both *in vitro* and *in vivo*

To ascertain that SCF^βTrCP^ promotes CDK1 ubiquitination, we carried out both *in vitro* and *in vivo* assays. First, we examined the *in vitro* ubiquitination of transcribed and translated ^35^S-labeled CDK1 by SCF^βTrCP^ (Fig 1J). In the presence of ubiquitin, high molecular weight ubiquitinated derivatives of CDK1 were formed (third lane), while βTrCPΔF prevented ubiquitination. SCF^FBXW7α^ was used as a selectivity control of CDK1-ubiquitin ligation for SCF^βTrCP^. These data indicate that SCF^βTrCP^ directly ubiquitinates CDK1, at least, *in vitro*. Next, we immunoprecipitated Flag-CDK1 in the presence of ubiquitin and βTrCP (or βTrCPΔF). We detected ubiquitinated forms of CDK1 with an anti-ubiquitin K48 specific antibody, which recognizes K48-linked polyubiquitin chains as mediators of proteasomal degradation, or an anti-ubiquitin K63 specific antibody, which binds to K63-linked polyubiquitin chains, that act in a range of other processes [13] (Fig 1K). In both cases, CDK1 was ubiquitinated by SCF^βTrCP^.

### Ubiquitin-mediated lysosomal degradation of CDK1

**Figure 2:**
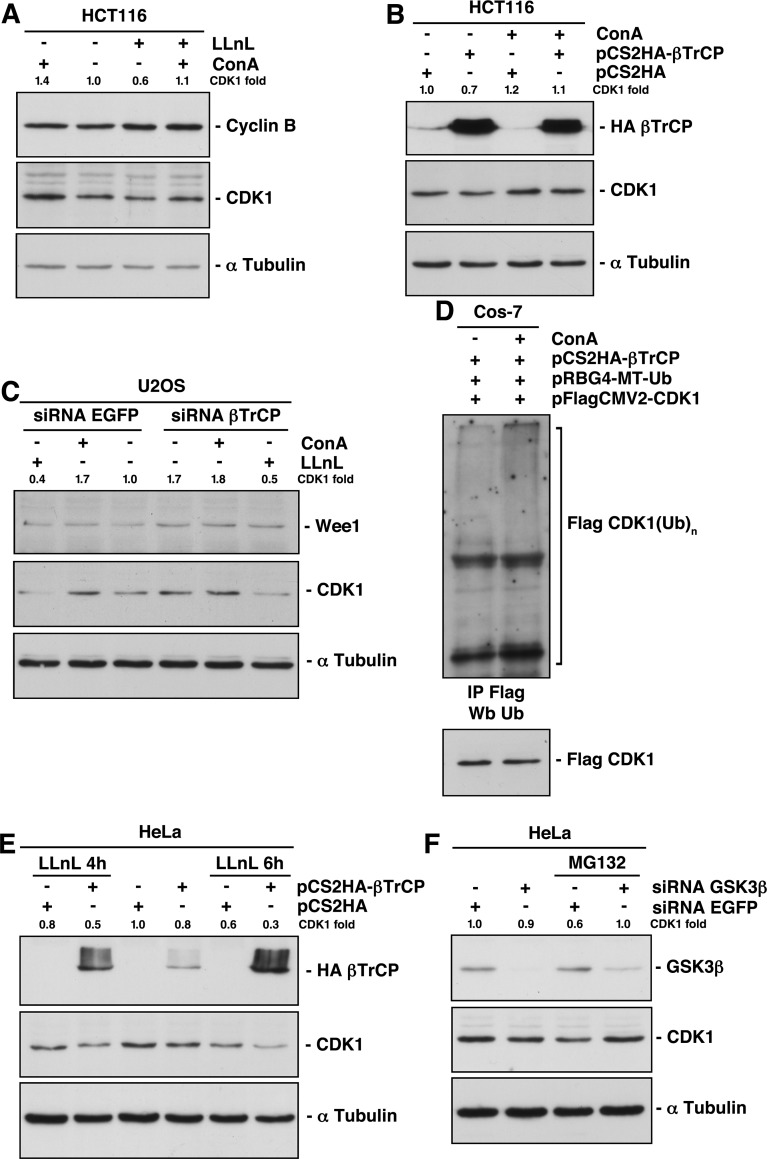
Lysosomal-mediated CDK1 degradation involves both SCF^βTrCP^-dependent and independent mechanisms (A) HCT116 cells were treated with proteasomal (LLnL) or/and lysosomal (ConA) inhibitors for 4 hours and protein expression analyzed by immunoblot. (B) HCT116 cells were transfected with the indicated plasmids and treated or not with ConA 4 hours before harvesting. Total extracts were blotted with different antibodies. (C) U2OS cells were interfered and treated with LLnL or ConA 4 hours before harvested. Extracts were analyzed by immunoblot. (D) Comparison of CDK1 ubiquitination from transfected cells treated or not with ConA. Flag-CDK1 was immunoprecipitated and blotted with the indicated antibodies. (E, F) HeLa cells were transfected or interfered and treated with proteasome inhibitors. Extracts were analyzed by Western blot. The quantitative fold change in CDK1 was determined relative to the loading control.

Eukaryotic cells utilize two major routes to effectively target a wide range of proteins for degradation: the ubiquitin/proteasome system and the autophagy/lysosome pathway [14]. To determine whether one or both of these systems were involved in the βTrCP regulation of CDK1 stability, we tested the effect of LLnL and concanamycin A (ConA) on CDK1 stability and their influence when cells were transfected or depleted of βTrCP. We found that ConA, a specific lysosomal inhibitor, increased the amount of CDK1. Unexpectedly, the proteasomal inhibitor LLnL, not only did not increase, but decreased CDK1 below control levels (Fig 2A). In fact, CDK1 was internalized to discrete autophagic vesicles within the cytoplasm after LLnL treatment (supplementary Fig S2A). Moreover, ConA partially prevented this reduction (Fig 2A, fourth lane), indicating that treatment with LLnL induced lysosomal degradation of CDK1. As a control, we analyzed the behavior of cyclin B, which was not affected by ConA and increased with LLnL treatment. The proteasomal inhibitors’ effect on CDK1 was confirmed in all cell lines tested and using two different inhibitors (supplementary Fig S2B). In addition, treatment with ConA substantially avoided the CDK1 degradation caused by βTrCP overexpression (Fig 2B). Furthermore, the increase of CDK1 induced by depletion of βTrCP was comparable to that caused by the treatment with ConA (Fig 2C, fourth vs. second lanes), and the latter could not enhance CDK1 levels in βTrCP interfered cells (Fig 2C, fourth vs. fifth lanes). These results strongly support the idea that βTrCP induces the degradation of CDK1 through the lysosome. This conclusion was validated by comparing the appearance of ubiquitinated forms of CDK1 promoted by βTrCP overexpression in the presence or absence of ConA (Fig 2D). Taken together, we have demonstrated that CDK1 ubiquitination induced by SCF^βTrCP^ provokes its lysosomal degradation.

### GSK3β mediates CDK1 degradation induced by proteasome inhibition

We previously described how inhibition of the proteasome by LLnL treatment decreased CDK1 levels (Fig 2A). The degradation of CDK1 induced by LLnL could not be reversed by βTrCP depletion (Fig 2C, first vs. sixth lanes). Similarly, the presence of LLnL in cells overexpressing βTrCP not only does not reduce the CDK1 degradation, but further increases it (Fig 2E). This is because SCF^βTrCP^ is not involved in the proteasomal mediated degradation of CDK1, and because LLnL protects βTrCP itself from degradation by the proteasome (compare the HA-βTrCP expression of the fourth lane with that of second and sixth lanes of Fig 2E).

A recent report provides evidence supporting a role of GSK3β in the regulation of autophagy activation in response to proteasome inhibition [15]. To investigate whether GSK3β mediates CDK1 degradation after LLnL treatment, we interfered HeLa cells and found that GSK3β depletion impedes the lysosomal destruction of CDK1 (Fig 2F). Therefore, these results reveal that GSK3β signaling determines a lysosomal βTrCP-independent degradation of CDK1 induced by proteasome inhibition.

### Identification of a βTrCP-binding site in CDK1

**Figure 3:**
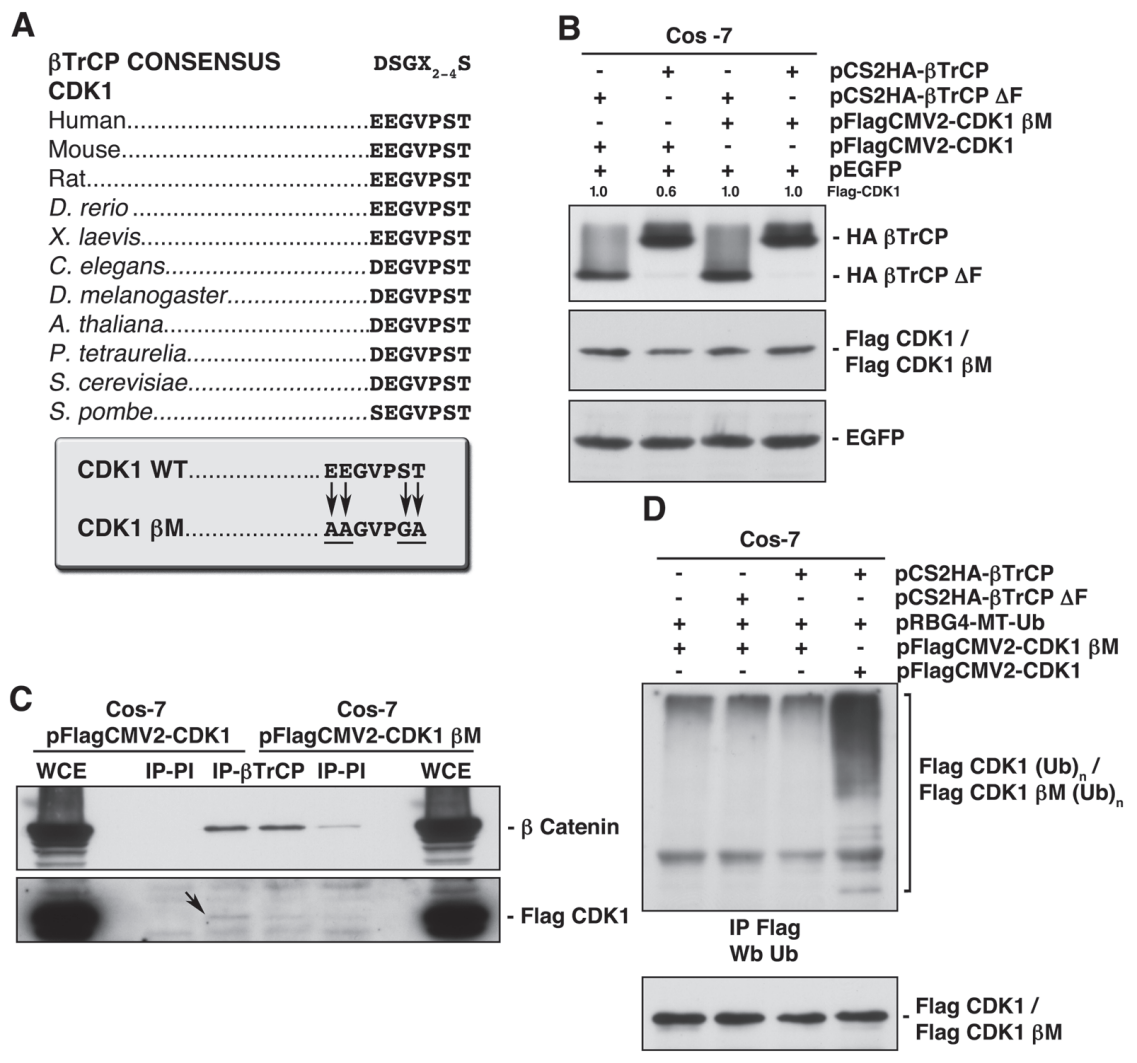
Identification of the βTrCP recognition motif in CDK1, whose mutation avoids both βTrCP/CDK1 interaction, and CDK1 ubiquitination and degradation (A) Alignment of the putative βTrCP consensus sequence identified in human CDK1 (residues 41 to 46) with other orthologs. Arrows indicate the amino acid changes performed to obtain a CDK1 mutated in this motif (CDK1 βM, CDK1 βTrCP mutant). (B) Cos-7 cells were transfected with the indicated plasmids and extracts analyzed by immunoblot. (C) βTrCP was immunoprecipitated from Cos-7 transfected cells, and coimmunoprecipitation of Flag-CDK1 or Flag-CDK1 βM studied with anti-Flag. Anti-β catenin was used as a control of βTrCP immunoprecipitation. IP-PI: immunoprecipitation with normal rabbit serum. WCE: whole cell extracts. (D) Cos-7 cells were transfected with the indicated plasmids, and Flag-CDK1 or Flag-CDK1 βM ubiquitination obtained after Flag immunoprecipitation and Western blot using anti-ubiquitin. The quantitative fold change in Flag-CDK1 was determined relative to the loading control.

Sequence analysis of CDK1 revealed the sequence EEGVPS, at residues 41-46, which is evolutionary conserved (Fig 3A). This sequence is very similar to the phosphodegron motif (DSGXXS) recognized by βTrCP, especially considering that phosphoserine may be replaced by an acidic amino acid (glutamic or aspartic acids) [16]. We generated a CDK1 mutant in its βTrCP-binding motif (CDK1-βM) by mutating residues 41, 42 and 46 to Ala or Gly. In addition, we mutated Thr47, next to the phosphodegron, since the consensus motif sometimes has from two to four amino acids of any type (X_2-4_) [17]. To verify that the CDK1-βM was insensitive to βTrCP-mediated degradation, we overexpressed both βTrCP and βTrCPΔF proving that neither degraded CDK1-βM (Fig 3B). βTrCP was unable to associate with (Fig 3C) nor ubiquitinate *in vivo* to the mutated version of CDK1 (Fig 3D). Given these data, we have identified the binding site of βTrCP to CDK1.

### DNA damage regulates βTrCP-induced CDK1 degradation in a cell type-dependent manner

**Figure 4:**
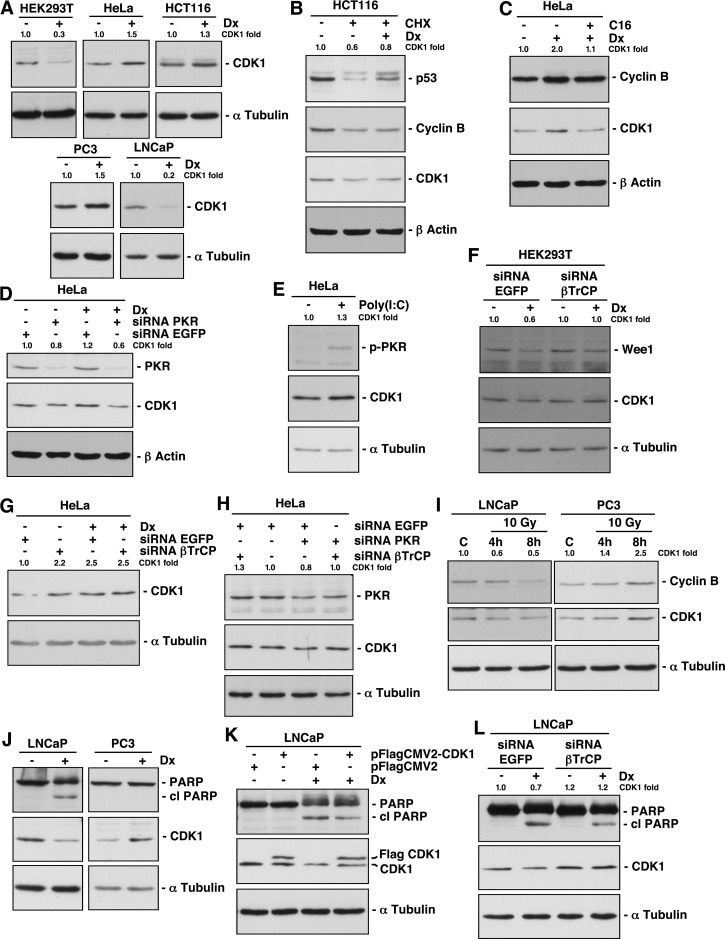
DNA damage alters βTrCP-induced CDK1 degradation and its corresponding induction of apoptosis in a cell type dependent manner (A) Cell lines were treated with doxorubicin (Dx) for 24 hours, and CDK1 expression analyzed by immunoblot. (B) Protein synthesis of HCT116 cells was arrested with cycloheximide (CHX) and treated or not with Dx for 10 hours. Extracts were analyzed by Western blot. (C) HeLa cells were treated with Dx for 24 hours and with C16, a PKR inhibitor, 2 hours before harvested. Proteins expression was studied with different antibodies. (D) HeLa cells were interfered with the indicated siRNAs, and 24 hours before harvested, treated with Dx. Extracts were blotted with the indicated antibodies. (E) HeLa cells were treated with poly(I:C), a PKR activator, for 6 hours, and extracts were analyzed by immunoblot. p-PKR indicates phospho-PKR (Thr451). (F, G) HEK293T and HeLa cells were treated as in (D). (H) HeLa cells were interfered with the indicated siRNAs and extracts analyzed by immunoblot. (I) LNCaP and PC3 cells were γ-irradiated to a dose of 10 Gy and harvested 4h and 8h later. Extracts were blotted with the indicated antibodies. C: control. (J) LNCaP and PC3 cells were treated as in (A). cl PARP: cleaved PARP. (K) LNCaP cells were transfected with the indicated plasmids and treated or not with Dx for 24 hours before harvested. Extracts were analyzed by Western blot. cl PARP: cleaved PARP. (L) LNCaP cells were treated as in (D). cl PARP: cleaved PARP. The quantitative fold change in CDK1 was determined relative to the loading control.

Since a previous study reported that CDK1 was downregulated upon genotoxic stresses [12], we studied the role of βTrCP on CDK1 stability after DNA damage. To this end, several cells lines were treated with doxorubicin for 24h and extracts analyzed by Western blot. We found that CDK1 stability depended on the cell types: CDK1 decreased in HEK293T and LNCaP cells, and increased in HCT116, HeLa and PC3 cells (Fig 4A). This behavior does not depend on the accumulation of cells in a specific phase of the cell cycle because the CDK1 amount is constant throughout the cell cycle [11]. In addition, doxorubicin treatment led to a decline in the G1:G2/M ratios in all cell lines, as expected (supplementary Fig S3) [18]. To eliminate the potential transcriptional effect of doxorubicin, before treating cells, protein synthesis was blocked with cycloheximide. Figure 4B shows that CDK1 protein from HCT116 cells was more stable when cells were treated with doxorubicin than when left untreated. Similar results were obtained for p53. Therefore, in HCT116 cells, DNA damage protects CDK1 from degradation. To inquire into mechanisms underlying CDK1 stability after DNA damage, we studied whether PKR was involved in CDK1 regulation, as previously suggested. Treatment with C16, a PKR inhibitor, or a specific PKR siRNA, both prevented the increase of CDK1 and even caused a reduction of CDK1 levels, after doxorubicin treatment (Figs 4C and 4D). Moreover, activation of PKR with poly(I:C) induced an increase of CDK1 (Fig 4E). In conclusion, the doxorubicin effect on CDK1 is mediated by PKR, both in cells where CDK1 increases (Fig 4) as in cells where decreases (supplementary Fig S4A, and [12]).

We next employed HeLa cells as a model in which CDK1 increases after doxorubicin treatment, and HEK293T, in which CDK1 decreases in order to study whether βTrCP was implicated in CDK1 stability after DNA damage. Knockdown experiments showed that βTrCP avoided the CDK1 diminution after doxorubicin in HEK293T cells (Fig 4F). However, the siRNA βTrCP-induced increase of CDK1 in HeLa cells was not augmented by treatment with the drug, suggesting that the effect of doxorubicin is produced through the loss of function of βTrCP (Fig 4G). This hypothesis was confirmed by the fact that the reduction of CDK1 obtained by siRNA PKR in HeLa cells was prevented in the absence of βTrCP (Fig 4H). Taken together, we can conclude that SCF^βTrCP^ mediates CDK1 stability after DNA damage.

### Implication of CDK1 degradation in the induction of apoptosis after DNA-damage

As we have demonstrated above, CDK1 is degraded in LNCaP and increased in PC3 cells after doxorubicin treatment. We were interested to study whether gamma irradiation induced similar results, because it is also used for the treatment of a wide variety of cancers, with probably less side effects. Figure 4I shows that both cell lines behave in the same way following irradiation as after doxorubicin treatment. A number of studies have established a correlation between CDK1 activity and apoptosis induced by DNA damage [19]. In fact, the CDK1 inhibitor RO-3306 induced apoptosis in all cell lines tested (supplementary Fig S4B). Thus, we analyzed whether CDK1 degradation was related to the onset of apoptosis. For this, we examined the appearance of the 85 kDa-cleaved fragment of PARP protein as a hallmark of apoptosis. We found that doxorubicin treatment induced PARP cleavage and degradation of CDK1 in LNCaP but not in PC3 cells, suggesting that both events could be associated (Fig 4J). To validate this result, we overexpressed CDK1 in LNCaP cells and found a significant PARP cleavage reduction after doxorubicin (Fig 4K). Similar results were observed using HEK293T cells (supplementary Fig S4C). Furthermore, βTrCP depletion in doxorubicin treated LNCaP cells, not only avoided CDK1 degradation as expected, but also diminished PARP cleavage (Fig 4L). Hence, we can deduce that βTrCP-mediated CDK1 degradation intervenes in apoptosis induced by DNA-damage.

### CDK1 accumulation in human tumors shows inverse correlation with βTrCP expression

**Figure 5:**
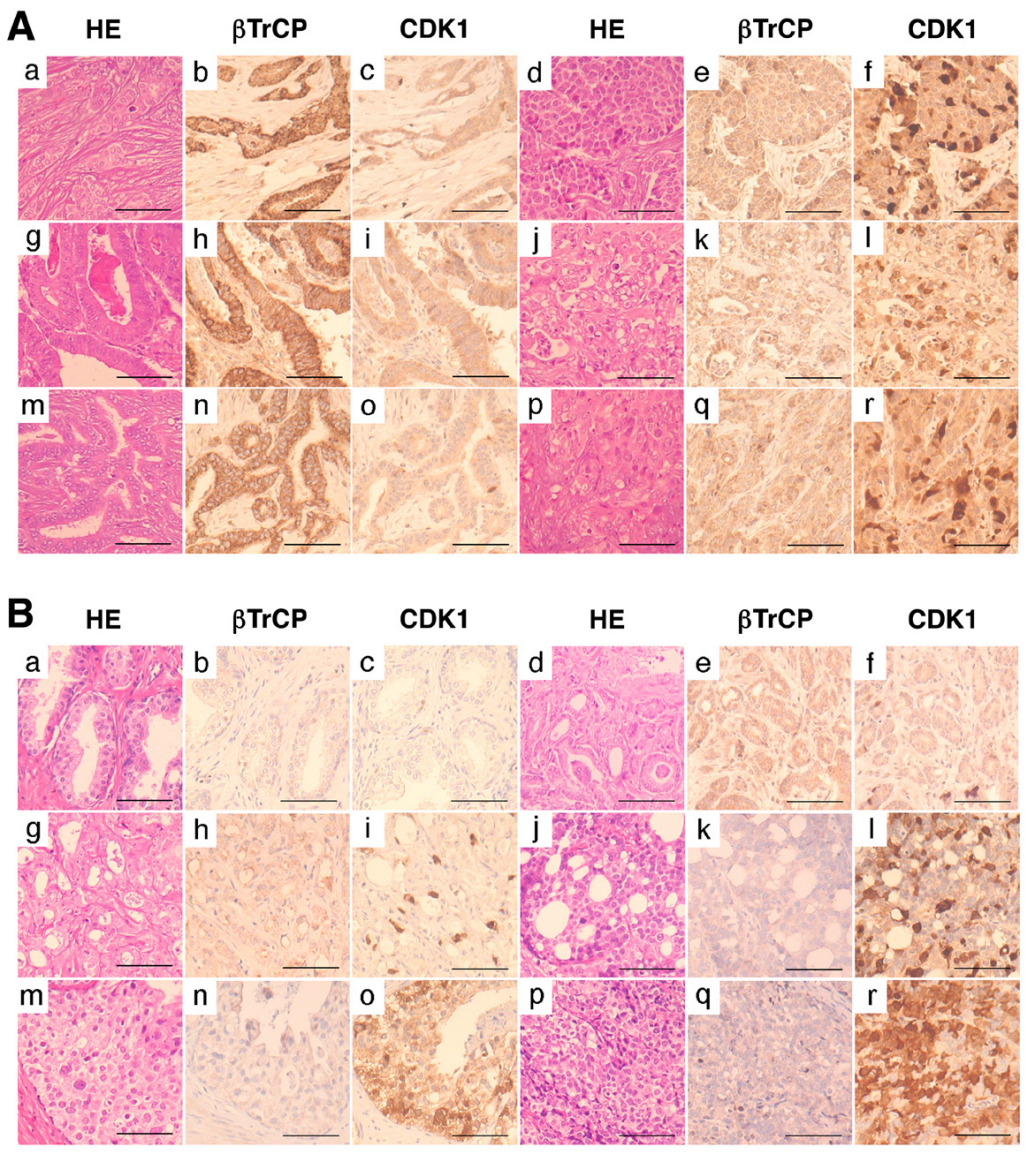
CDK1 accumulation in human tumors shows inverse correlation with βTrCP expression and is associated with tumor grade (A) Immunohistochemical expression of βTrCP and CDK1 in human breast carcinomas (a-f), gastric carcinomas (g-l), and cholangiocarcinomas (m-r). Bars, 200μm. (B) Immunohistochemical expression of βTrCP and CDK1 in benign prostate glands (a-c), low grade prostatic carcinomas (d-i), and high grade prostatic carcinomas (j-r). HE, hematoxylin-eosin. Bars, 200μm.

According to our results, CDK1 expression in tumors could be modulated by βTrCP expression and have an impact on the degree of malignancy of tumors. To check this, we first analyzed the immunohistochemical expression of CDK1 and βTrCP in a tissue microarray containing 66 human carcinomas of different sites. Forty three (65.1%) carcinomas showed high levels of CDK1. Of these 43 tumors, 40 showed reduced (low to moderate) levels of βTrCP, while 13 out of 23 tumors with low CDK1 showed high βTrCP expression. On the other hand, 50 (75.7%) carcinomas showed reduced levels of βTrCP. Of these 50 tumors, 40 expressed high CDK1 levels, and conversely, CDK1 expression was reduced in 13 out of 16 carcinomas with high βTrCP. Representative immunostains are shown in Figure 5A. Inverse correlation between CDK1 and βTrCP expression was statistically significant in this series of tumors (p<0.05, from Fisher's exact test). The fact that high CDK1, low βTrCP phenotype was seen in a majority of tumors may reflect the proportion of high grade carcinomas in this tissue microarray. To further address this, we analyzed the accumulation of CDK1 in 109 carcinomas of the prostate (60 low grade, 49 high grade) and looked for its association with tumor grade. Fifty eight (96.7%) low grade carcinomas showed low levels of CDK1, while 39 (79.6%) high grade carcinomas expressed elevated levels of CDK1. Representative immunostains are shown in Figure 5B. Association of CDK1 accumulation and tumor grade was statistically significant in this series of prostatic cancers (p<0.05, from Fisher's exact test). These results suggest that loss of βTrCP may facilitate CDK1 accumulation in tumors, particularly in those of high grade of malignancy.

## DISCUSSION

It has been shown that *CDK1* transcription/translation oscillates in a cell cycle-specific manner [11]. However, CDK1 levels are constant during the normal cell cycle. Therefore, degradation of the protein must be strictly regulated. We have demonstrated that SCF^βTrCP^ contributes to maintain invariable the CDK1 level throughout the cell cycle by its lysosome-mediated degradation. Moreover, proteasome inhibition further induced the lysosomal degradation of CDK1, in which GSK3β signaling was involved. However, recently Yoon *et al.* reported that CDK1 increased after MG132 treatment [12]. The reasons for this discrepancy are unclear, but we confirmed CDK1 degradation after proteasome inhibition in the seven cell lines tested.

**Figure 6:**
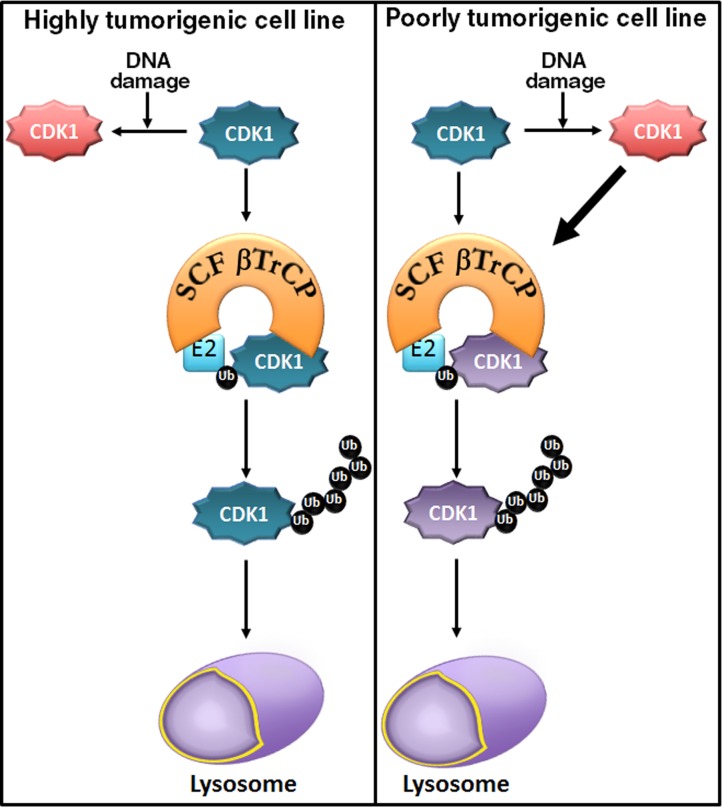
A model of the mechanism of CDK1 degradation In all cell lines tested, CDK1 (in blue) is ubiquitinated and degraded by SCF^βTrCP^/lysosome in control conditions (basal phosphorylation status). In highly tumorigenic cell lines, DNA damage inhibits CDK1 degradation (in red), whereas in poorly tumorigenic cell lines it enhances CDK1 degradation (thick black arrow). CDK1 (in purple) indicates both CDK1 from control conditions and after DNA damage. Ub (in black): ubiquitinated CDK1.

DNA damage induces cell cycle arrest through several mechanisms [20]. In regard to CDK1, damage triggers stabilization of inhibitory phosphorylation sites on CDK1 [21] and repression of *CDK1* gene expression [22]. Here, we present an additional novel mechanism that involves CDK1 polyubiquitination by SCF^βTrCP^ and its degradation by the lysosome. This effect appears to depend on the cell type, since in some cell lines DNA damage induces CDK1 degradation, whereas in others it prevents destruction of the protein, probably due to changes in the folding of CDK1. Although more studies are required to confirm this, the difference could reflect the distinct tumorigenic ability of the diverse cell lines. Thus, HEK cells have been reported to have a moderate tumorigenic potential, being used as a cellular model for normal human cells to study the oncogenic potential of a number of genes [23, 24]. LNCaP cells are non-invasive and poorly tumorigenic, whereas PC3 cells are highly tumorigenic and invasive [25], and similarly HeLa and HCT116 are highly tumorigenic [26, 27]. Figure 6 shows a model of the mechanism of CDK1 degradation in accord with our results.

It has been published that PKR phosphorylates amino-terminal Tyr4 of CDK1 inducing its ubiquitination in cells where CDK1 is degraded after doxorubicin treatment [12]. In fact, we have found that PKR modifies the stability of CDK1 mediated by SCF^βTrCP^/lysosome after DNA damage. We have identified the βTrCP-binding site in CDK1 between residues 41 to 46. How Tyr4 phosphorylation may alter the ubiquitination/degradation induced by βTrCP is a matter that remains to be elucidated. Perhaps, CDK1 is folded in such a way that its phosphorylation changes the βTrCP access to the molecule allowing or not its ubiquitination and degradation, as has been previously suggested [12].

Furthermore, in this study we reveal that βTrCP-mediated CDK1 degradation is associated with genotoxin-mediated apoptosis. Thus, doxorubicin-mediated apoptosis in LNCaP cells was attenuated by CDK1 overexpression or by βTrCP depletion. In concurrence with our results, it has been reported that PKR mediates the inhibition of cell proliferation and apoptosis under stress conditions in which CDK1 is degraded under such conditions [12, 28]. More importantly, cells where CDK1 was not degraded after DNA-damage did not induce PARP cleavage and, consequently, did not progress into apoptosis. These data are of particularly high impact, given that by a simple analysis of biopsy samples from patients treated with genotoxins or gamma radiation, a read-out could be obtained as to whether tumors will suffer apoptosis or recurrence.

Finally, in different tumor types from patient samples, we showed an inverse correlation between CDK1 accumulation and βTrCP levels. We also observed a positive correlation between CDK1 accumulation and the degree of malignancy of tumors. *CDK1* gene amplification or overexpression occurs in human tumors, but our results also suggest that loss of βTrCP may facilitate CDK1 accumulation. These results support the development of molecules that promote the degradation of CDK1 as a future therapeutic target for cancer treatment.

## METHODS

### Plasmids, cloning, point mutation and sequencing

pCS2HA-βTrCP, pCS2HA-βTrCPΔF and empty vectors have been previously described [29-31]. pEGFP-N1 and pRBG4-MT-Ub were from BD Biosciences and ATCC, respectively. pFlagCMV2-CDK1 and pCMVHA-FBXW7α were obtained by cloning the corresponding PCR fragments in pFlagCMV2 and pCMVHA, respectively. Flag-CDK1 βM was constructed using the “Transformer Site-Directed Mutagenesis Kit” from BD Biosciences. Construct sequences and point mutations were verified on both strands with an automatic sequencer.

### Cell culture, cell synchronization, transient transfections, drugs and γ-irradiation

Routinely, HeLa, HCT116, Cos-7, U2OS, HEK293T, PC3 and LNCaP (from ATCC), and HCT116 *p53^−/−^* cells [32] were grown in Dulbecco's modified Eagle's medium (Lonza) as described [33]. Cells enriched in the different phases of the cell cycle were also obtained as previously described [34], or by treating them with butyrate (6mM), aphidicolin (APH 1μM), RO-3306 (RO 9μM) or nocodazole (NZ 10μM), all from Sigma, and confirmed by flow cytometry. DNA constructs were transiently transfected by electroporation or using lipid transfection reagents (Lipofectamine (Invitrogen) or Xfect (Clontech)), and 18h or 48h post-transfection, respectively, cells were harvested and lysed. For some experiments, cells were treated with Ac-LLnL-CHO (LLnL 100μM, Sigma), MG132 (MG 20μM, Boston Chemical), concanamycin A (ConA 50nM, Sigma), doxorubicin (Dx 1μM, Sigma), C16 (1μM, Merck), poly(I:C) (500ng/ml, Sigma) or cycloheximide (CHX 50μg/ml, Sigma), and harvested at various times. Where indicated, cells were γ-irradiated to a dose of 10 Gy and harvested 4h and 8h later.

### Subcellular fractionation and lysis

Subcellular fractionation was carried out as described [35]. Whole cell extracts were prepared at 4°C in 420mM NaCl, 10mM Tris-HCl (pH 7.5), 1% Nonidet P-40 (NP40), 10% glycerol, 1mM PMSF (phenylmethylsulfonyl fluoride), 1μg/ml aprotinin, 1μg/ml pepstatin, 1μg/ml leupeptin and 10μg/ml chymostatin for 20min. Extracts were centrifuged at 20,000 g for 20min and supernatants frozen in liquid nitrogen and stored at -80°C. Protein concentration was determined using the Bradford assay (Bio-Rad). After DNA damage, cell extracts were treated with lambda protein phosphatase (λ-PP) [36].

### Small interfering RNA (siRNA) assays

Cells were interfered with βTrCP1/2-, GSK3β- or PKR-siRNA [37-39] using the Oligofectamine method (Invitrogen) to suppress the expression of endogenous genes. EGFP-siRNA [38] was used as a non-specific control. Cells were harvested 48h post-transfection and reduction of protein levels confirmed by Western blotting.

### Electrophoresis, Western blot analysis and antibodies

Proteins were separated by SDS-polyacrylamide gel electrophoresis (SDS-PAGE) and gels were electroblotted onto nitrocellulose membranes and probed with the following antibodies: anti-HA-peroxidase monoclonal antibody (Roche); anti-CDK1 and anti-p53 monoclonal antibodies (Santa Cruz); anti-α-Tubulin, anti-Flag and anti-β-actin monoclonal antibodies (Sigma); anti-GFP polyclonal antibody (Immune Systems Ltd.); anti-WEE1 and anti-K48 and anti-K63-linkage specific polyubiquitin polyclonal, and anti-ubiquitin monoclonal antibodies from Cell Signaling; anti-phosphoPKR monoclonal antibody (Epitomics); and anti-cyclin B, anti-β-catenin, anti-GSK3β, anti-PARP and anti-PKR monoclonal antibodies (BD Biosciences). Peroxidase-coupled donkey anti-rabbit IgG and sheep anti-mouse IgG were obtained from GE Healthcare. Immunoreactive bands were visualized using the Enhanced Chemiluminescence Western blotting system (ECL, GE Healthcare).

### Co-immunoprecipitation experiments

Whole cell extracts diluted to 150mM NaCl (1-2 mg) were incubated with normal rabbit serum for 30 minutes and subsequently with protein A-sepharose beads (GE Healthcare) for 1 hour at 4°C. After centrifugation, beads were discarded and supernatants incubated for 2 hours with anti-βTrCP rabbit monoclonal antibody (Cell Signaling) or normal rabbit serum, followed by protein A-sepharose beads for 1 hour. Beads were washed and bound proteins were solubilized by the addition of SDS-sample buffer heated at 95°C for 5 minutes.

### Tandem mass spectrometry (MS/MS)

HA-βTrCP was transfected into Cos-7 cells. After 18h, cells were treated for 4h with LLnL and HA-βTrCP immunoprecipitated from nuclear and cytosolic fractions with anti-HA (Covance) previously coupled to a resin from Pierce (AminoLink Plus Coupling Resin). Resins were washed three times in NP-40 lysis buffer and six times in ammonium bicarbonate 50mM. Samples were analyzed by MS/MS using a LTQ mass spectrometer (Thermo Electron) as described previously [40].

### *In vitro* and *in vivo* ubiquitination assays

The *in vitro* ubiquitination of CDK1 was performed in a volume of 10μl containing 50mM Tris-HCl (pH 7.6), 5mM MgCl_2_, 0.6mM DTT, 2mM ATP, 2μl *in vitro* transcribed/translated unlabeled F-box protein, 1.5ng/μl E1 (His_6_-ubiquitin activating enzyme, Boston Biochem), 10ng/μl His_6_-UbcH3 (E2, Boston Biochem), 10ng/μl UbcH5a (E2, Boston Biochem), 2.5μg/μl ubiquitin (Sigma), 1μM ubiquitin aldehyde (Boston Biochem), and 1μl ^35^S-methionine-labelled *in vitro* transcribed/translated CDK1 as substrate. The reactions were incubated at 30ºC for 1h and analyzed by SDS-PAGE and autoradiography.

The *in vivo* ubiquitination experiments were performed in Cos-7 cells transfected and treated with ConA 4 hours before harvesting. Cells were washed in PBS, lysed at 95ºC for 15 minutes in NP40 buffer supplemented with 5% SDS and 10mM iodoacetamide, and diluted in NP40 buffer supplemented with 10mM iodoacetamide. Flag-CDK1 was immunoprecipitated and proteins separated by SDS-PAGE, electroblotted and probed with different antibodies.

### Immunofluorescence microscopy

HeLa cells were grown on coverslips, treated or not with LLnL for 4 hours, and fixed with 4% paraformaldehyde and permeabilized with 0.1% Triton X-100. Immunostaining, using monoclonal anti-CDK1 (Santa Cruz) and polyclonal anti-LC3 (Novus Biologicals) antibodies, and counterstaining with DAPI to visualize the nuclei, was carried out according to standard protocols. Epifluorescence microscopy was performed using a Leica microscope.

### Tumor immunohistochemistry

Two tissue microarrays, one containing 66 human carcinomas of different sites (breast (6), prostate (5), liver (3), stomach (4), pancreato-biliary (6), colorectal (4), lung (10), head and neck (4), kidney (4), bladder (4), ovary (5), cervix (3), endometrium (2), and thyroid (6), and one with 109 carcinomas of the prostate, were used for immunohistochemistry. All the carcinoma tissues (1-mm cores) were collected from archival paraffin blocks at this hospital, with the approval of the institutional ethical board. After dewaxing, sections were subjected to heat-induced epitope retrieval in 10mM EDTA pH 9.0, and were incubated with either anti-βTrCP rabbit polyclonal antibody (1:150; Cell Signaling) or anti-CDK1 rabbit polyclonal antibody (1:400; Santa Cruz) overnight at 4ºC. Peroxidase-labeled secondary reagents, chromogen (diaminobenzidine), and hematoxylin counterstain were applied according to manufacturer's protocols (Dako). Immunostaining was evaluated by two observers and scored as low (<10%), moderate (10-25%), or high (>25%), according to the percentage of immunostained cells. Moderate and high categories were combined as positive for the purpose of statistical analysis. Prostate tumors were classified into low grade or high grade according to Gleason score, the latter being tumors with Gleason score 4+3 or higher. Statistical analyses were performed on contingency tables using Fisher's exact test. Two-sided p value <0.05 was considered as significant.

### SUPPLEMENTARY MATERIAL FIGURES


