# Supplementary material for: βTrCP controls the lysosome-mediated degradation of CDK1, whose accumulation correlates with tumor malignancy
Source: Oncotarget. 2014 Jul 27;5(17):7563–74. doi: 10.18632/oncotarget.2274 (PMC4202144; doi:10.18632/oncotarget.2274)
Supplement: Supplementary file 1 [file oncotarget-05-7563-s001.pdf]

βTrCP controls the lysosome-mediated degradation of CDK1, whose accumulation correlates with tumor malignancy

Supplementary Material

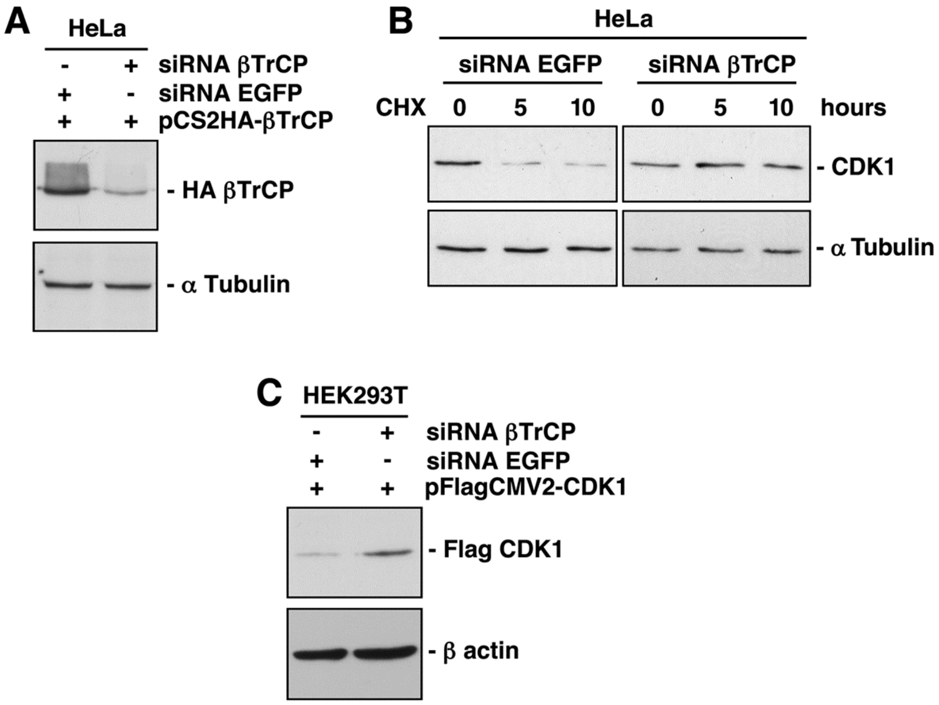

Figure S1: Effect of βTrCP depletion on the total amount of CDK1. (A) HeLa cells were transfected with pCS2HA-βTrCP and interfered with siRNA-βTrCP to check the HA-βTrCP depletion. Total extracts were analyzed by immunoblot. (B) HeLa cells were interfered with EGFP- or βTrCP-siRNA and, after 48h, cycloheximide (CHX) was added to the medium and cells were collected at the indicated times. Extracts were analyzed by Western blot. (C) HEK293T cells were transfected with pFlagCMV2-CDK1 and interfered with siRNA-βTrCP. The amount of Flag-CDK1 was measured by Western blot.

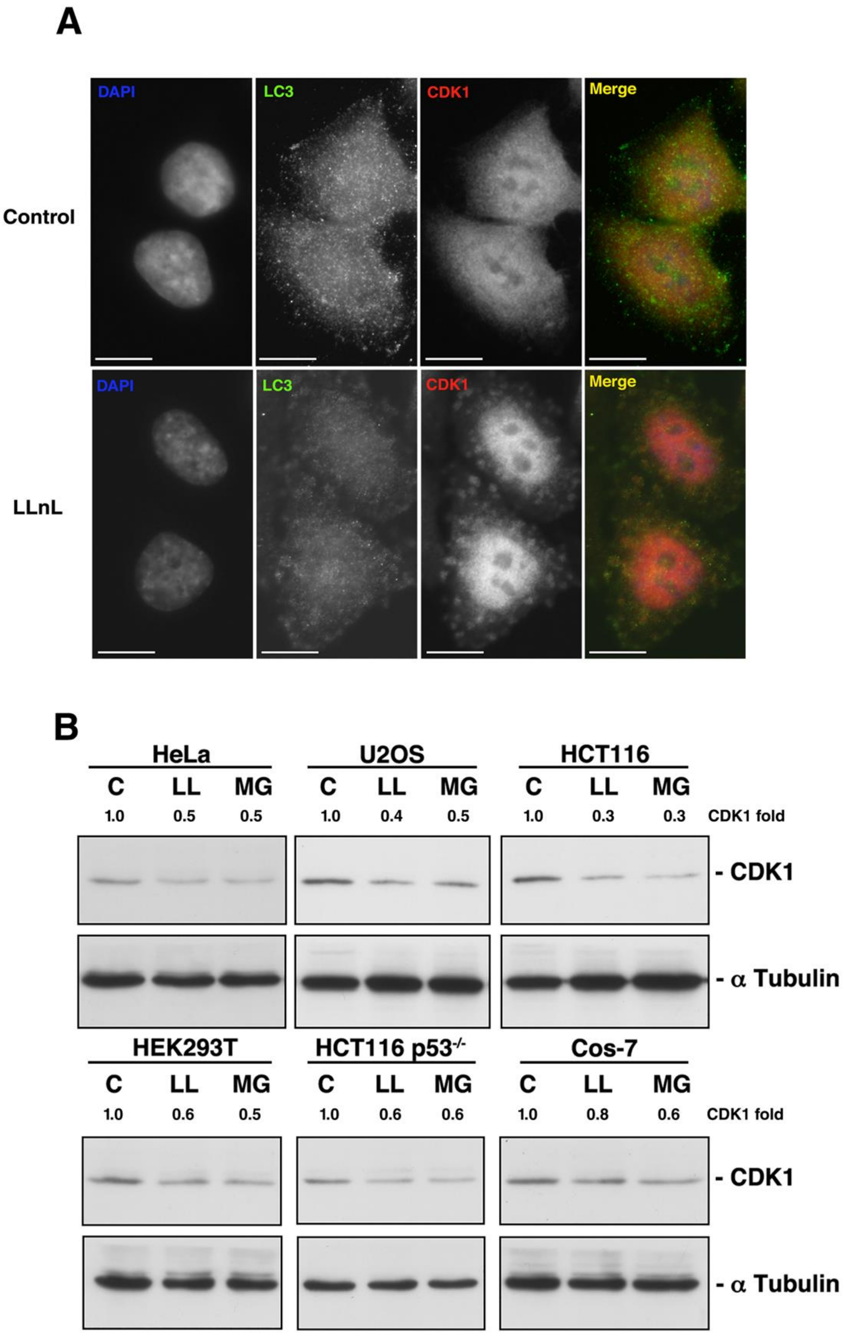

Figure S2: Effect of the proteasome inhibition on CDK1. (A) The autophagic marker light chain 3 (LC3) forms a shell around CDK1 after LLnL treatment. HeLa cells treated or not with LLnL for 4 hours were stained for CDK1, LC3 and DNA. In the merge, CDK1 staining is shown in red and LC3 in green. Bars, 10μm. (B) Several cell lines were treated with LLnL (LL), MG132 (MG) or control (C) for 4 hours and extracts analyzed by immunoblot. The quantitative fold change in CDK1 was determined relative to the loading control.

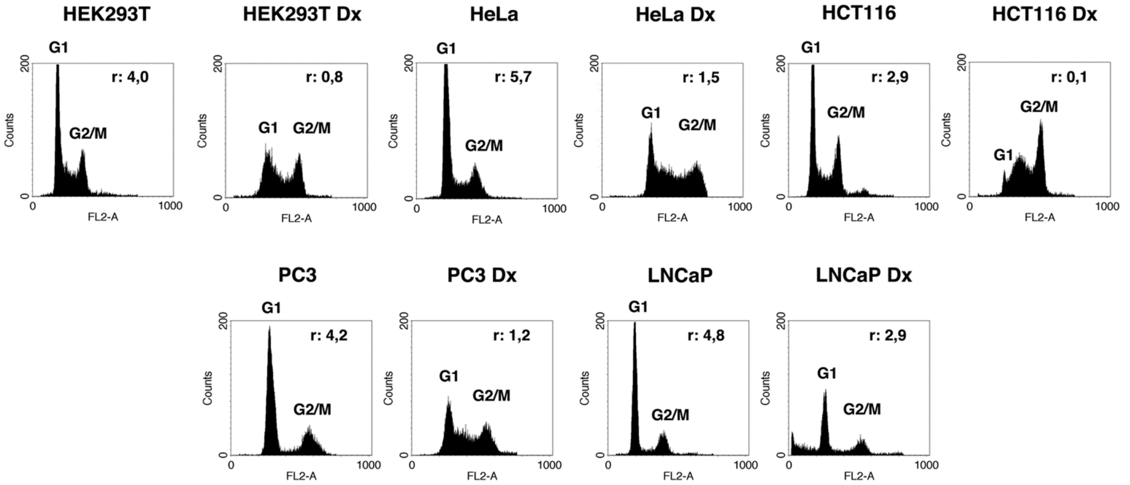

Figure S3: Effect of doxorubicin on cell cycle. Different cell lines were treated or not with doxorubicin and cell cycle analyzed by flow cytometry. r: G1:G2/M ratio.

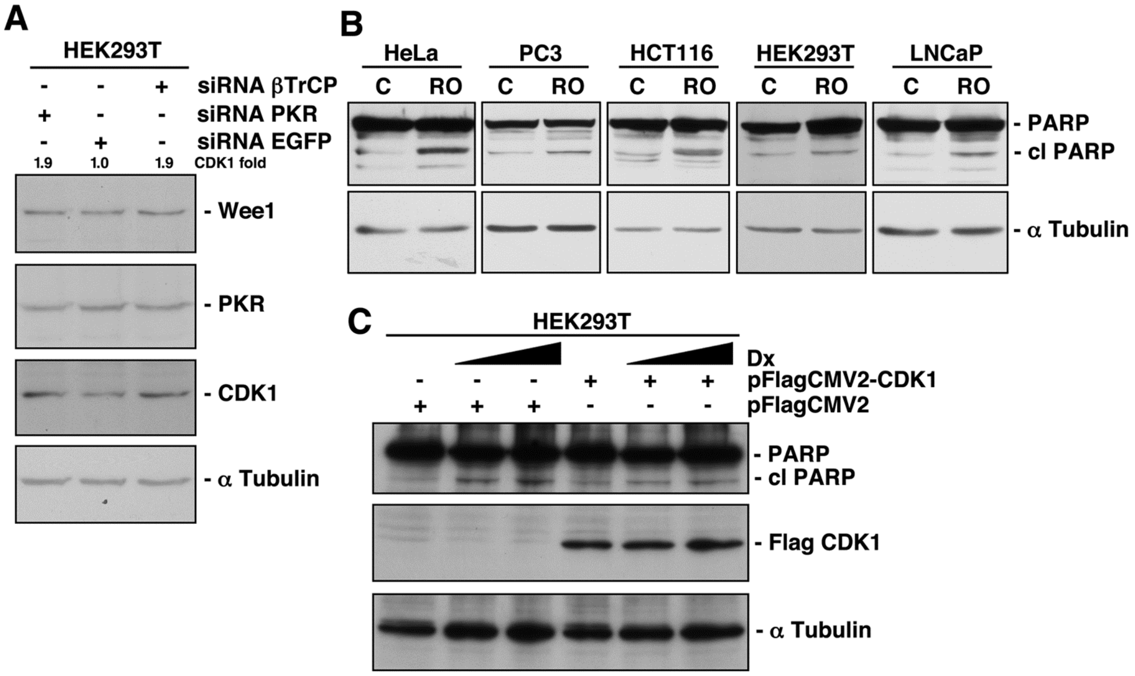

Figure S4: PKR and βTrCP mediate CDK1 degradation in HEK293T cells, and CDK1 overexpression in these cells reduced PARP cleavage after doxorubicin treatment. (A) HEK293T cells were interfered with the indicated siRNAs and extracts analyzed by Western blot. (B) Different cell lines were treated (RO) or not (C) with the CDK1 inhibitor RO-3305 for 24 hours and extracts analyzed by Western blot. cI PARP: cleaved PARP. (C) HEK293T cells were transfected with the indicated plasmids and treated with two concentrations of doxorubicin (Dx) for 24 hours before harvested. Extracts were blotted with different antibodies. cl PARP: cleaved PARP. The quantitative fold change in CDK1 was determined relative to the loading control.
